# Fracture Toughness Analysis of Epoxy-Recycled Rubber-Based Composite Reinforced with Graphene Nanoplatelets for Structural Applications in Automotive and Aeronautics

**DOI:** 10.3390/polym12020448

**Published:** 2020-02-14

**Authors:** Alaeddin Burak Irez, Emin Bayraktar, Ibrahim Miskioglu

**Affiliations:** 1 LMT—Laboratoire de Mécanique et Technologie, Université Paris-Saclay, ENS Paris-Saclay, CNRS, 94235 Cachan, France; 2 School of Mechanical and Manufacturing Engineering, SUPMECA-Paris, 93400 Saint Ouen, France; bayraktar@supmeca.fr; 3 ME-EM Department, Michigan Technological University, Houghton, MI 49931, USA; imiski@mtu.edu

**Keywords:** toughening mechanisms, graphene nanoplatelets, recycled rubber, Halpin–Tsai, SEM

## Abstract

This study proposes a new design of lightweight and cost-efficient composite materials for the aeronautic industry utilizing recycled fresh scrap rubber, epoxy resin, and graphene nanoplatelets (GnPs). After manufacturing the composites, their bending strength and fracture characteristics were investigated by three-point bending (3PB) tests. Halpin–Tsai homogenization adapted to composites containing GnPs was used to estimate the moduli of the composites, and satisfactory agreement with the 3PB test results was observed. In addition, 3PB tests were simulated by finite element method incorporating the Halpin–Tsai homogenization, and the resulting stress–strain curves were compared with the experimental results. Mechanical test results showed that the reinforcement with GnPs generally increased the modulus of elasticity as well as the fracture toughness of these novel composites. Toughening mechanisms were evaluated by SEM fractography. The typical toughening mechanisms observed were crack deflection and cavity formation. Considering the advantageous effects of GnPs on these novel composites and cost efficiency gained by the use of recycled rubber, these composites have the potential to be used to manufacture various components in the automotive and aeronautic industries as well as smart building materials in civil engineering applications.

## 1. Introduction

Over the past decades, aeronautic companies have been continuously trying to reduce the overall cost and mass of an aircraft to better compete with their rivals. Mass reduction leads to lower fuel consumption and CO_2_ emissions. Therefore, development of low-cost and lightweight materials to be used in the manufacture of various aircraft parts constitutes an important task for engineers in aeronautic companies.

In aircrafts, polymer-based composites are used widely in the manufacturing of various structural and functional components including the wings, tail, and skin panels. In material selection, structural requirements are, of course, important. Also, the cost of the material should be minimized without compromising the structural requirements. To that end, proposing a low-cost, lightweight aircraft material was the main objective of this study.

Polymer-based composites are extensively used to manufacture lightweight structural components. Including recycled materials in the production may result in cost efficiencies as well as ecological solutions. Epoxy, a thermosetting polymer, which is relatively easy to process and has low cost, is often used as the matrix material in the polymer-based composites. Although epoxies have high stiffness and specific strength and are environmentally stable [1], they are brittle due to the fact of their highly cross-linked network structure. Therefore, to improve the toughness of epoxy, secondary phase particles, such as soft (thermoplastic particles, rubber) and rigid fillers, are added [2,3,4]. Hence, the use of recycled rubber in the proposed composites can meet the expectations on toughness and material cost.

The disposal of used or scrap rubber parts poses a technical, ecological, and economic challenge due to the fact of their vulcanized structure [5]. For instance, discarded tires in a landfill can hold water, creating habitats for mosquito larvae as well as other animals such as rodents and snakes [6]. These sites can potentially be sources for diseases such as malaria, cephalitis, dengue, and chikungunya. Besides, if the rubber piles in the landfills burst into flames, it is difficult to extinguish [7]. In addition, some additives in the rubber discarded at landfills, such as colorants, stabilizers, flame retardants, and plasticizers, may leach into the soil and cause further ecological problems [8,9,10]. Using recycled rubber in composite manufacturing can help to reduce the impact of discarded rubber on the environment with the added benefit of cost reduction. Various research groups have reported studies on recycled rubber-epoxy blends. As the first example, a research group used recycled rubber to modify epoxy resin to improve its toughness, with minimal change on strength and stiffness. In their study, it is proposed that the manufactured material can be used to manufacture railroad cross ties in high volumes [11].

Another research group argued that the blends of epoxy and recycled rubber can be used in agricultural areas for cementing the adhesive bonding of larger units. In addition, the same material can serve to fill larger cracks and to shape imbalances [12]. Also, due to the quite inhomogeneous cross-linked structure of rubbers and rubber-like materials, recycled rubber-modified epoxies can be utilized as sound and vibration dampers [13,14,15,16].

Apart from the positive outcomes, it is claimed that recycled rubber particles significantly decrease the shear strength of the overlapped adherent because the boundary of the particles and the resin promote the formation of cracks, decreasing the strength [17]. In order to compensate for the decrease in strength, hybridization of the composite by fillers, such as graphene nanoplatelets (GnPs), offer an optimum composition as a bracket material to be used in aircraft wings [10,18,19].

Nano graphene has unique properties as a result of its 2D honeycomb structure which makes it a promising nanoscale inclusion for polymer nanocomposites. It has an outstanding mechanical strength (130 GPa) and specific elasticity modulus (1 TPa) [20,21,22]. Besides, the high surface area of the nano fillers can improve the properties of the composites even at very low contents compared to the microscale fillers [23,24,25]. Also, as an added benefit, high electrical conductivity of graphene can reduce the risk of damage to aircrafts from lightning strikes.

In the frame of this research, after manufacturing these novel composites, three-point bending (3PB) tests were carried out to determine the fundamental mechanical properties, and the results were compared with FEM modelling and with a modified Halpin–Tsai homogenization adapted to GnP-consisting composites. In addition, composite fracture toughness was examined using notched specimens. Lastly, fracture surfaces were observed with a scanning electron microscope (SEM) to study the toughening and damage mechanisms.

## 2. Experimental Procedure

### 2.1. Materials

In this study, graphene nanoplatelets, recycled EPDM (ethylene propylene diene monomer) rubbers, and epoxy matrix were used to manufacture the specimens. Graphene nanoplatelets were procured from Alfa Aesar™ with the specific name of “Graphene nanoplatelets aggregates, sub-micron particles, S.A. 500 m^2^/g”. This product consists of sub-micron platelets which have a diameter of less than 2 microns and a thickness of around 5 nanometers. The tensile modulus and the density of the of the GnPs were listed as 1 TPa and 2.25 g/cm^3^, respectively. For the matrix, Araldite DBF epoxy resin and its hardener Aradur HY 956 EN were obtained from Hunstman™. Araldite DBF has a tensile modulus of 2880 MPa and a density of 1.1 g/cm^3^. The hardness of Araldite DBF is given as 80 in Shore D scale for 25 °C. Recycled EPDM rubber was supplied by a sports equipment manufacturer in Sofia, Bulgaria, as fresh scrap, i.e., they were collected directly from the production line as waste parts and pulverized. No contaminants were found in the rubber such as metallic particles which could oxidize and overheat the rubber or degrade the adhesion of rubber with the matrix. The average diameter of the rubber particles was measured as 10.44 μm by a Cilas™ 990 Laser Particulate Analyzer. The modulus of elasticity of the EPDM rubber was provided by the recycled rubber supplier as 6 MPa, elongation at break was 80–100%, its hardness was 37–40 Shore A, and the density was 1.4 g/cm^3^.

### 2.2. Materials Processing and Experimental Characterization

**Figure 1:**
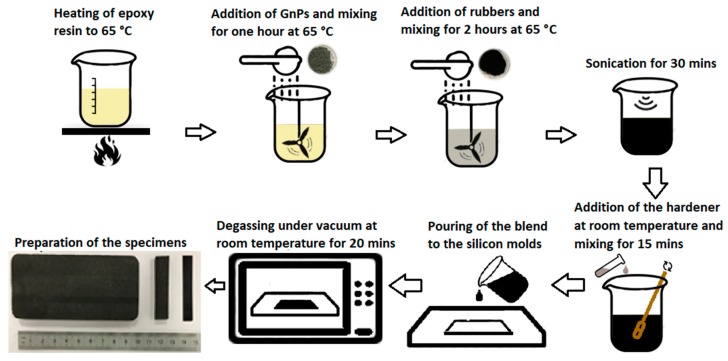
Manufacturing flow chart for the recycled ethylene propylene diene monomer (EPDM)-modified epoxy-based composites.

The manufacturing process of the composites is illustrated in Figure 1, and more details can be found in our previous paper [4]. Sonication was performed to distribute GnPs and rubber particles more homogeneously in the epoxy matrix. Degassing of the molded final composite was required to eliminate the air bubbles that may have generated during the polymerization of epoxy.

In this study, the content of rubber particles and GnPs added to epoxy were varied to investigate their effects on the mechanical properties of the composite. The compositions of the composites (referred to as LG, LR, and LRG composites hereafter) used in the study are given in Table 1.

The densities of the composites were measured with a pycnometer, and Shore D hardness measurements were carried out according to the ASTM D 2240 standard. Quasi-static three-point bending tests (3PB-Instron 5569, Norwood, MA, USA) were performed in accordance to the ASTM D790 standard. Load on the specimen and midspan deflection were measured during each test. Midspan deflection of the specimen was measured by the crosshead position. In addition, fracture toughness parameters, such as critical stress intensity factor (*K_Ic_*) and critical strain energy release rate (*G_Ic_*), were investigated with single-edge notched-beam (SENB) specimens according to the ASTM D5045 standard. At least five specimens for each composition were used. The SEM fractography (Scope/JSM-6010LA Jeol^®^, Tokyo, Japan) was performed on the fracture surfaces to identify the toughening and damage mechanisms.

## 3. Results and Discussion

### 3.1. Physical Characteristics of the Manufactured Composites

The measured densities of the LRG composites are given in Table 2.

As expected, composite densities increased with the increasing GnP content. Density of the ternary composites (LRG) were bracketed by the densities of the binary (LG and LR) composites. Moreover, in binary group composites (LR groups), increasing rubber content decreased the density of the composites. When this trend is compared with the Shore D hardness, it is observed that the hardness of the composites was reduced. The density reduction also lowered the mechanical performance of the binary composites. Strain at break and the maximum flexural stress suffered from the decreasing density in the LR groups. However, in the ternary group composites (LRG), the composite performance cannot be simply associated to the density. More intricate mechanisms are involved in the composite performance together with density.

Another physical characteristic of the composites, surface hardness, is presented in Table 3.

It can be seen that the addition of 0.5 wt. % GnP did generally enhance the composite hardness due to the fact of their hard nature compared to rubber and epoxy. However, there was no remarkable change in hardness when reinforcement contents were increased further to 1.0 wt. % and then to 1.5 wt. %. As GnPs have high affinity and strong van der Waals forces, they tend to agglomerate when their content is increased. For this reason, it is difficult to distribute GnPs homogeneously in the microstructure and have a proportional increase in the hardness.

### 3.2. Mechanical Characterization of the Manufactured Composites by Means of 3PB Tests

**Figure 2:**
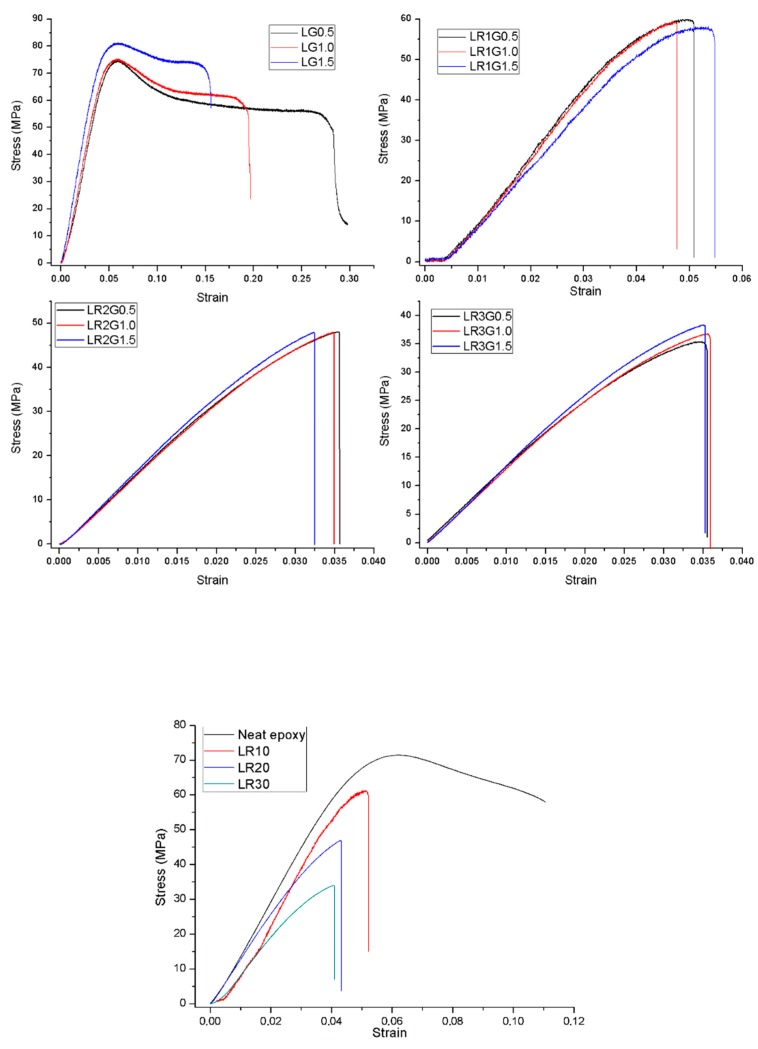
Engineering stress–strain curves of the manufactured composites.

The results from the three-point bending tests are given in Figure 2 for one sample from each composite group, and all results are summarized in Table 4 with their standard deviations.

Figure 2 shows that for LG composites (epoxy and GnPs), the strain at break increased with increasing GnP content, whereas for the LR composites (epoxy and rubber), the strain at break decreased with increasing rubber content. It is also observed in Table 4 that GnPs did not have a significant effect on the strength of the composites.

The increase in rubber content resulted in a drop in both the strength and strain at break of the composites. This tendency can be related to the poor interfacial adhesion of the recycled EPDM and epoxy blends. Because the EPDM rubber was vulcanized during the post-processing phase of its manufacturing cycle, it lacked free links on its surface. Thereby, the recycled EPDM lacked free links also, making it difficult to have a chemical bond with epoxy. Because of the incompatibility between recycled EPDM and epoxy, composite interfaces may contain some voids, and this gives rise to low stress transfer from the matrix to the rubber particles reducing the global rigidity of the compounds. Moreover, in the course of the solidification, different contractions of rubber and epoxy can bring some inequalities in the internal stress balance which leads to void formations at the composite interfaces. In addition, by the increasing content of the recycled rubber particles, the possibility to observe any agglomerations increases. Therefore, these agglomerations create the weak parts of the composite. As a consequence, when the composites are subjected to any loading, these abovementioned voids and agglomerations constitute the weak points of the composite where cracks can initiate. This state produces premature failure. Also, low rigidity of recycled EPDM has an effect in the drop of the mechanical properties of the composites.

The strain at break generally increased for 0.5–1.5 wt. % GnP loading for 10 wt. % recycled rubber content which indicates better chain mobility. However, with a further increase in recycled rubber loading as the number of particles increase, chain mobility as well as elongation at break decreased. The poor flexural strength of LR2G and LR3G group composites was mainly due to the inhomogeneous dispersion of recycled rubber filler in the epoxy matrix and the presence of GnPs agglomerates in the composite which inhibited stress transmission and reduced the flexural strength of the composite.

**Figure 3:**
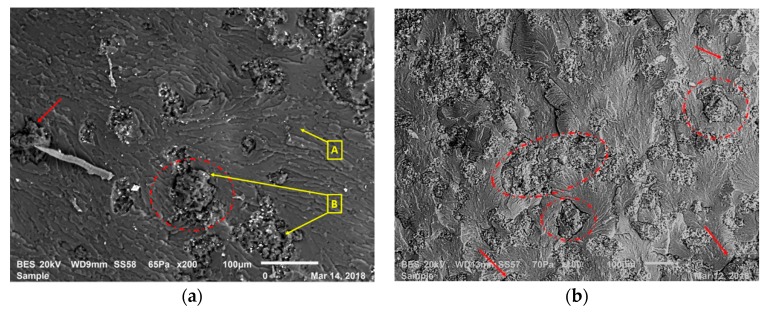
Comparison of the fracture surfaces: (**a**) 10% rubber-consisting composition—LR1G0.5; (**b**) 30% rubber-consisting composition—LR3G0.5.

These arguments are supported by the fracture surface images in Figure 3a,b, taken after the 3PB tests. Figure 3a indicates the fracture surface of a composition with 10 wt. % of rubber, whereas Figure 3b shows a composition with 30 wt. % rubber. In Figure 3b, the composite with 30 wt. % rubber content had more discontinuities, shown with the red circles and arrows, at the epoxy and rubber interfaces in comparison to the composite with 10 wt. % rubber content. In Figure 3a,b, rough areas show recycled rubber particles, whereas smooth areas indicate the epoxy matrix. In addition, in Figure 3b, compared to composites with lower rubber content, more agglomerates of rubber particles are observed. Because of the above factors, the increase in rubber content enhanced the amount of discontinuities, resulting in a reduction in strength and strain at break of the composites manufactured. Therefore, GnPs were introduced into the composite structure to compensate for these adverse effects of recycled rubber. The GnPs’ rigid nature enhanced the composites’ modulus of elasticity. However, the GnPs were not able to efficiently compensate for the mentioned adverse effects of recycled rubber for stress and strain at break [26,27].

In addition, the flexural moduli of the LR1G0.5 and LR1G1.5 groups indicate some deviations from the expected values. This can stem from the issues related to composite manufacturing. For instance, during the mixture process, rubber particles and GnPs can aggregate in the microstructure, and these may create stress concentrations resulting in lower mechanical properties. Also, these composites are cured with a specific hardener at a predefined temperature. In this curing process, cross-links are created along the polymer chains. In general, if the density of the cross-links increases, the cured composite becomes more rigid. In this regard, during the curing procedure, some points, at the micro scale, can locally be cured either insufficiently or excessively. Therefore, these local differences may constitute the weak or strong points of the composites which influence the final mechanical properties. Apart from these anomalies, addition of GnPs improved the flexural modulus of the 20–30 wt. % recycled rubber containing epoxy blends. On the other hand, increasing rubber content degraded the flexural modulus of the binary epoxy recycled rubber composites ($E_{LR10}>E_{LR20}>E_{LR30}$).

### 3.3. Numerical Verification of the 3PB Tests

In this step of the study, experimental results were compared with numerical approaches. In this context, a modified Halpin–Tsai model considering the effects of nanoplatelets was used as the homogenization strategy. In the modified Halpin–Tsai model, shape factor and aspect ratio of the inclusions were taken into consideration. Otherwise, incorrect shape factor adoption may lead to erroneous results. The modified Halpin–Tsai model is given in Equations (1) and (2) [28,29]:

(1) $$E_{c}=\left(\frac{3}{8}\;\frac{1+\left(2L_{G}/3T_{G}\right)\;\eta_{L}V_{G}}{1-\eta_{L}V_{G}}+\frac{5}{8}\frac{1+2\eta_{T}V_{G}}{1-\eta_{T}V_{G}}\right)E_{P}$$

(2) $$\eta_{L}=\;\frac{\left(E_{G}/E_{P}\right)-1}{E_{G}/E_{P}+2L_{G}/3T_{G}}\;\eta_{T}=\;\frac{\left(E_{G}/E_{P}\right)-1}{E_{G}/E_{P}+2}$$

In these equations, *E_C_* is the elasticity modulus of the final composite with randomly oriented graphene nanoplatelets, and *E_G_* and *E_P_* are the elasticity moduli of graphene and the matrix, respectively. The modulus of elasticity of the matrix was reckoned as the combination of the EPDM rubber particles with epoxy according to studies in the literature [2]. *V_G_*, *T_G_*, and *L_G_* refer to the volume fraction, thickness, and length of graphene. The dimensions of the graphene sheets were provided by the supplier of the GnPs. On the other hand, to estimate the modulus of elasticity of the epoxy–recycled rubber blend, classical H–T equations were used, as below:

(3) $$\frac{E_{m2}}{E_{matrix}}=\frac{1+\xi \eta \varphi_{R}}{1-\xi \eta \varphi_{R}},\;\eta =\frac{\frac{E_{R}}{E_{matrix}}-1}{\frac{E_{R}}{E_{matrix}}+\xi },$$

Here, *E_m2_*, *E_R_*, and *E_matrix_* are the modulus of elasticity of the epoxy–rubber blend, recycled rubbers, and the epoxy matrix, respectively, and *E_R_* was taken as 6 MPa. *φ*_R_ is the volume fraction of the rubber particles, the shape factor *ξ* of the rubber particles was assumed to be 2 (spherical particles).

**Figure 4:**
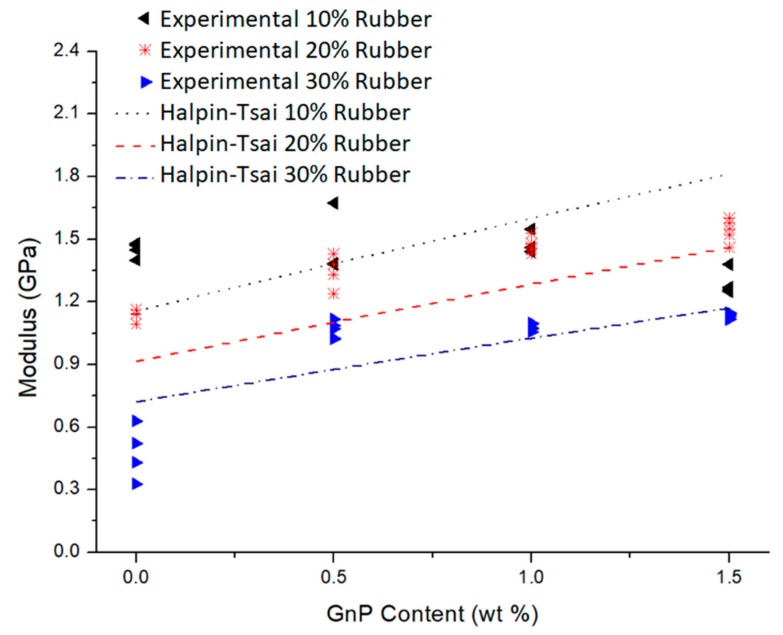
Experimental results and Halpin–Tsai model comparison of elasticity modulus of LRG composites by the increasing content of GnPs.

The moduli of the composites estimated according to Equations (1)–(3) are presented in Figure 4 along with the experimental results.

Figure 4 shows that the moduli of elasticity of the ternary composites (LRG groups) obtained by the Halpin–Tsai model had a reasonable agreement with the experimental results. In particular, the Halpin–Tsai model had better agreement with the experimental results for 20–30 wt. % rubber-containing composites. In general, for the compositions of LR2G and LR3G groups, the Halpin–Tsai model underestimated the moduli of elasticity. The synergistic effect of GnPs and rubber at the higher contents may be a reason for getting higher moduli experimentally than that predicted by the Halpin–Tsai model. The unanticipated decline in the experimental results is attributed to agglomeration of GnPs and recycled rubber particles at the higher contents.

After using the Halpin–Tsai for the homogenization, 3PB tests were simulated under the same test conditions by using the Abaqus™ FEM solver. These FEM calculations were performed mainly to observe the stress field of the composites subjected to bending moments as well as to plot the numerically obtained stress–strain curves to compare with the experimental ones. Numerical simulations were only implemented for the LR2G1.5 and LR3G1.5 group compositions, as the modified Halpin–Tsai modelling yielded estimates for the moduli that compared satisfactorily with the experimental results. In these simulations, material properties such as density, obtained from different characterization methods were introduced to the FEM solver, and the tests were simulated by dimensions of the specimens used in the simulations which were the same as the actual specimens used in the 3PB experiments. Boundary conditions were also implemented as in the 3PB tests. The displacements leading to the failure of the specimens (displacement at break) were imposed on the specimens in Abaqus™ as in the 3PB tests. In Table 5, the fundamental characteristics of the numerical calculations in Abaqus are given. After running the calculations in Abaqus™, results were obtained and compared with the experimental results.

**Figure 5:**
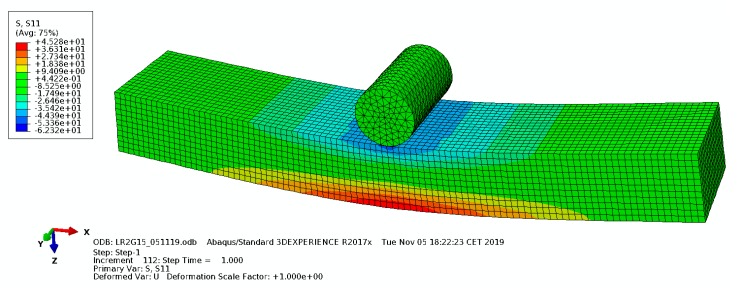
Stress field obtained from the finite element method (FEM) analysis for LR2G1.5.

The stress field obtained from the FEM analysis for the LR2G1.5 specimen under 3PB loading is given in Figure 5. As expected, at midspan, at the bottom of the specimen maximum tensile stress was observed. This stress leads to crack initiation followed by crack propagation and eventually to the failure of specimen.

**Figure 6:**
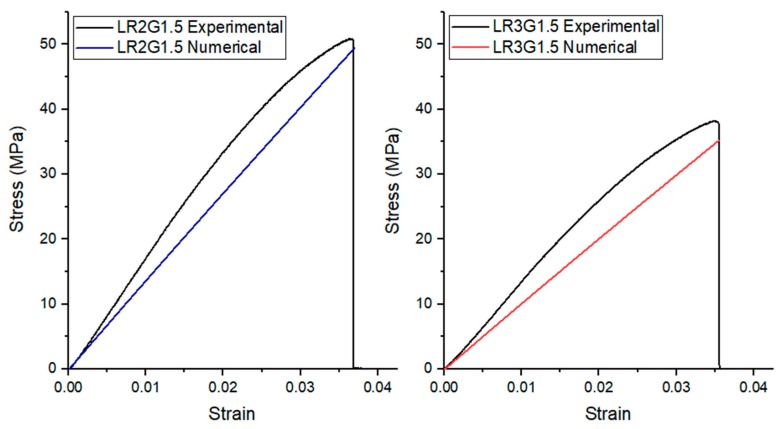
Comparison of the experimental results and FEM analyses.

Figure 6 depicts the experimental and numerical comparison of the stress–strain curves for the LR2G1.5 and LR3G1.5 group specimens. A reasonable estimate of the experimental results were observed by using Halpin–Tsai homogenization in this numerical modelling. The lack of yielding on the stress–strain curves indicates the brittle failure of the composites. In addition, the non-linearity in the curve can be associated to a combination of interfacial slippage and the expansion of plasticity in the matrix. By this way, the energy absorption capability can be increased while preserving the high stiffness of the composite [30].

### 3.4. Determination of the Fracture Toughness and Toughening Mechanisms Identification by Means of SEM

**Figure 7:**
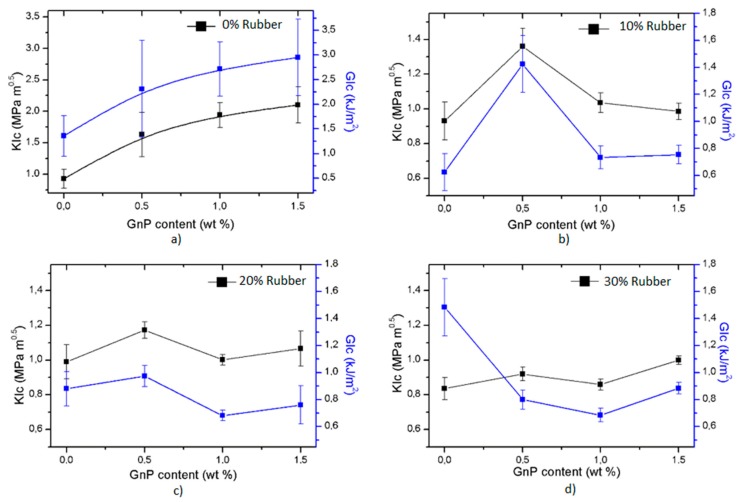
Variation of fracture toughness with GnPs for (**a**) 0% rubber, (**b**) 10% rubber, (**c**) 20% rubber, (**d**) 30% rubber.

Due to the manufacturing and post-processing methods or during service, cracks or defects may occur in the materials. Also, some discontinuities or particles with undesired geometries inside the microstructure can behave as cracks, and they can be responsible for the fracture of materials. Therefore, it is not always the most reliable way to design critical aeronautical part, such as the brackets in the wings, by considering the yield strength of the manufactured composites, since fractures may occur in the presence of cracks at smaller loads. For this reason, using fracture mechanics is considered the best option to get through such problems [31]. Fracture toughness is a numerical designation of the resistance of material to crack propagation under load. After performing bending tests using SENB-type notched specimens, the fracture toughness (*K_Ic_*) and fracture energy (*G_Ic_*) of the manufactured composites are given in Figure 7.

In Figure 7a, the favorable impact of GnPs on fracture toughness of the epoxy resin is quite apparent. As explained in detail in the following sub-section, toughening mechanisms, including GnPs layer separation and crack deflection, can increase the fracture toughness of the epoxy resin. Moreover, increasing the content of GnPs improved the *K_Ic_* of these binary composites. In addition, fracture energy (*G_Ic_*) is a second order function of *K_Ic_* $\left(\left(G_{Ic}=\;\frac{K_{Ic}{}^{2}\left(1-v^{2}\right)}{E}\right)\right)$ *v*: Poisson’s ratio, E: Modulus of Elasticity), and this explains the similar trend of *G_Ic_* to *K_Ic_*. However, this quadratic form of *G_Ic_* leads to larger error bars in Figure 7.

The variation of *K_Ic_* and *G_Ic_* was not as smooth for the epoxy–recycled rubber blends with GnPs (Figure 7b–d). As explained before, epoxy–recycled rubber blends may contain many discontinuities in the microstructure. Moreover, it is challenging to distribute GnPs uniformly in the epoxy matrix (high affinity of carbon atoms and van der Waals forces) which can influence the fracture toughness of the composites. Therefore, it is not surprising to observe fluctuations in the *K_Ic_*-*G_Ic_* of the epoxy–recycled rubber and GnP-containing ternary composites [32,33,34].

Lastly, in Figure 7d, for 30 wt. % rubber content, the relative magnitudes of *K_Ic_* and *G_Ic_* were different than the 10 and 20 wt. % rubber. This is due to the fact that the elastic modulus of LR30 was much lower than the elastic moduli of LR10 and LR20.

After mechanical tests, the fracture surfaces of the 3PB test specimens were observed via SEM. From these observations, various toughening mechanisms were determined.

**Figure 8:**
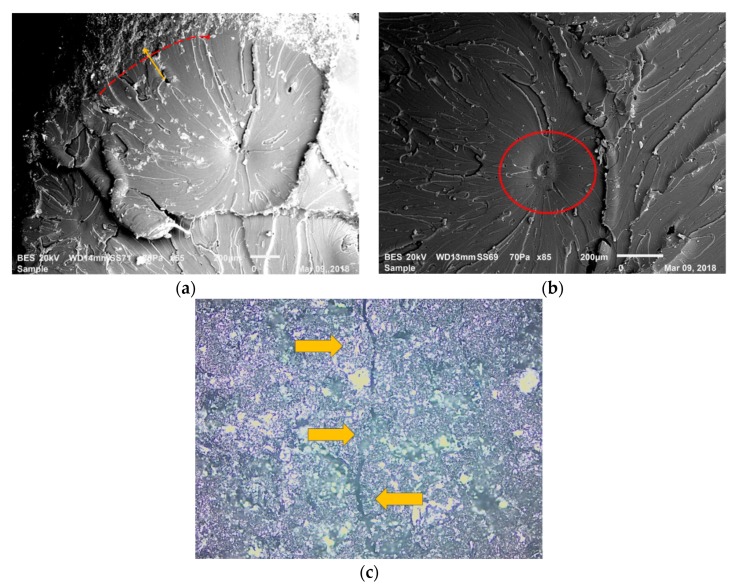
Fracture surface observation in LRG group composites: (**a**) tortuous passage in the fracture of GnP-reinforced epoxy-based composites; (**b**) dimple generation; (**c**) crack deflection.

One toughening mechanism was observed as crack deflection. If a crack comes across a hard particle or a different form of a reinforcement during its propagation, it finds alternative paths to maintain its propagation. As a consequence, a wavy crack propagation line is observed as seen in Figure 8c. The GnPs in the epoxy matrix may behave as stress concentrators, and they generate many micro-cracks. These micro-cracks increase the total fracture surface area because of crack deflection [35]. Also, a height difference between the deflected crack front and the original crack front is observed once the crack is deflected. This leads to a tortuous passage and explains the rough surfaces observed in Figure 8a [36,37].

Moreover, shear forces during the crack propagation facilitate the separation of the graphene sheets. To separate the GnP sheets, a certain amount of energy needs to be consumed and this energy is provided from the fracture energy. If a certain quantity of fracture energy is consumed, the energy needed to propagate the crack will not be enough for total rupture. Therefore, this phenomenon improves the fracture toughness. Also, the crack deflection and GnP layer separation create a combined toughening mechanism. This combined mechanism generates a characteristic “dimple-type” fracture surface which is shown in Figure 8a,b [38,39]. These dimple-type fracture surfaces are also accepted as crack initiation sites.

**Figure 9:**
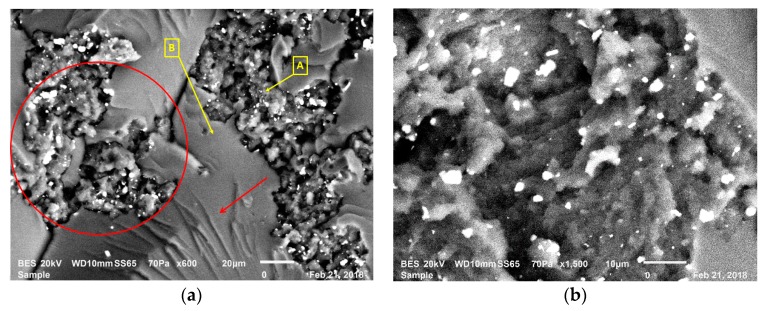
SEM fractography on the LRG group’s compositions: (**a**) shear band formation; (**b**) void observation.

Another toughening mechanism is shown in Figure 9a as shear yielding (indicated with shear bands marked with the red arrow). Also, inside the red circle in Figure 9a, cavitated and torn rubber particles surrounded by epoxy matrix can be observed. This encircled zone is magnified in Figure 9b, and the local rough surfaces in this figure show an enhanced area of fracture in these composites. This circumstance is an indicator for mechanisms of crack deflection and cavitation.

Many factors can lead to cavitation such as the resin system, curing pressure, environmental conditions. Hence, it is challenging to attain a void-free composite section. In the epoxy matrix, plastic deformation of the polymer and debonding of the inclusions can remarkably alter the number and the size of the voids formed. In this research, to modify the epoxy matrix, GnPs and recycled EPDM rubber were used. The GnPs and recycled rubber have high bulk moduli (K = E/3(1 – 2ν)). Because GnPs possess a high modulus of elasticity (E ≅ 1000 GPa), recycled EPDM rubber also has a Poisson’s ratio (*v*) of around 0.49. This states that GnPs and recycled EPDM particles are very rigid elastic bodies when they are subjected to triaxial stresses, and they are going to be highly resistant to any volumetric deformation. However, Poisson’s ratio of the epoxy matrix is approximately 0.33 which is substantially lower than rubber. Moreover, the epoxy matrix strain softens after yielding which is seen typically in the glassy polymers, and accordingly, the yielded epoxy matrix is going to be relatively compliant and will plastically deform more smoothly. However, the “rigid” rubbery phase and GnPs will obstruct any significant plastic dilatation in the matrix, unless these GnPs and rubber particles are pulled-out from the epoxy matrix or if the rubber particles do not internally cavitate. Thereby, by the interfacial debonding of GnPs and rubbers, some of the stored strain energy is dissipated and it is followed by shear yielding and shear band formation of the epoxy matrix encircling the fillers.

Shear yielding identified by shear bands is an important mechanism that enhances the strength of the polymer in the case of ductile fracture. Even though the final composite indicates a brittle fracture, crack propagation includes a localized viscoelastic and plastic energy dissipating process around the crack tip because of the polymer matrix. As a result, energy absorption is promoted by this mechanism in the material, and it enhances the material’s fracture toughness [40,41].

Following to debonding and shear yielding, cavitation arises in the epoxy matrix surrounding GnPs and rubber particles because of the localized plastic flow. Then, by the increasing the number of cavitations and plastic deformation of the epoxy matrix, some amount of the stored strain energy is dissipated which improves the composites’ fracture toughness [42,43,44,45].

**Figure 10:**
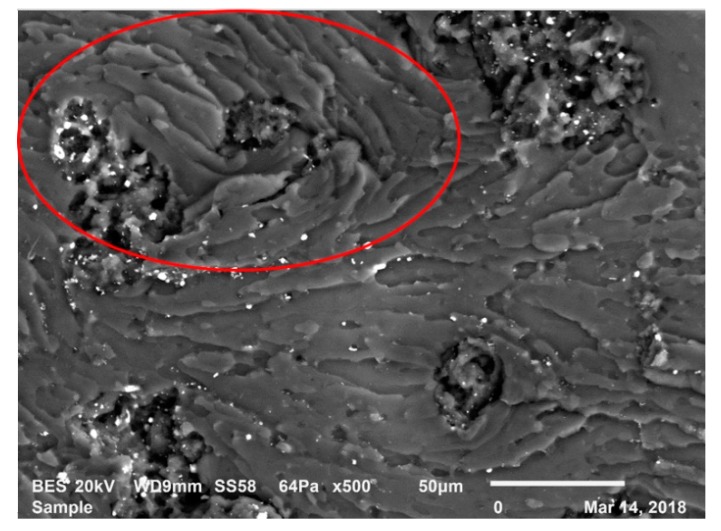
Ductile yielding in the LRG group’s compositions.

Lastly, when GnPs are found in front of the crack during crack propagation, twisted crack pathways are generated due to the GnPs as shown in Figure 10. In these regions, some of the fracture energy can be dissipated by the interaction of GnPs with the crack. In Figure 10, a lamellar structure (inside red ellipse) was observed as an indicator of the ductile yielding. This structure also indicates the transition between ductile and brittle states [46]. As a consequence, if there is ductile yielding, more energy can be dissipated during the fracture which increases the fracture toughness of the composites [47].

Fracture surface observation on the ternary composites showed different toughening mechanisms provided by GnPs and rubbers. The GnP layer separation and crack deflection were seen as the main mechanisms that improved the fracture toughness of the composites. In addition, cavitation and shear yielding were promoted by the presence of the recycled rubbers and GnPs. In conclusion, a good combination of different toughening mechanisms improved the fracture toughness of the manufactured composites.

The results presented in this study constitute a permitted part of an ongoing project with the aeronautic industry. The complete design of the considered element could not be presented in this manuscript in detail. The idea to use recycled rubber with the combination of epoxy and graphene nanoplatelets was presented in this manuscript. Based on the comparison of numerical and experimental results, the mechanical strength of the manufactured composites can be improved further by using more advanced manufacturing facilities enabling them to resist the applied loads more effectively. Therefore, these composites can provide a cost-efficient solution for aircraft manufacturers.

## 4. Conclusions

A solution blending method was used to manufacture epoxy-based novel composites for aeronautical applications. This method is a practical solution for manufacturing such composites at laboratory scale, and it can be scaled up to an industrial level easily. Increasing the rubber content brings a reduction in the density of the epoxy–recycled rubber blends, and this can be considered a positive outcome for the sake of the lightweight property of the composites. However, due to the interface issues and agglomerations, higher rubber rates can be avoided for the composites targeting mechanical performance. Otherwise, surface modifiers can be used on recycled rubbers to increase their affinity with the epoxy resin. In addition, the positive effect of GnPs on the fracture toughness and the elastic modulus was more apparent by the increasing rubber content. Therefore, GnP reinforcement becomes more reasonable for the increased rubber content.

Halpin–Tsai homogenization proposes a rapid estimation of the elasticity modulus of the ternary group composites, and it can be utilized to simulate the mechanical behavior of the composites in the elastic region.

It was seen that GnPs were involved in many toughening mechanisms which brought about significant improvement in the fracture toughness of the epoxy. In particular, the improvement in the mechanical performance with a very low content of GnP indicates the potential of this kind of composite.

By considering all the results in this study, these novel composites can be used in auxiliary components in the wings of an aircraft as well as luggage weather strip and radiator lining manufacturing in the automotive industry after eliminating issues with homogeneity. Moreover, manufactured composites may offer potential applications not only in aeronautics but also in the building engineering industry as smart building materials. One interesting hint should be given for aeronautic manufacturers here: the very high electrical conductivity of GnPs brings a new and original idea for the multifunctionality of these novel composites, and it may reduce the damage risk from lightning strikes.

## Figures and Tables

**Table 1:** Composition of Graphene nano platelets (GnP)-reinforced recycled rubber-blended epoxy-based composites.

LRG Composites	GnP Content (wt. %)			
Rubber content (wt. %)	0%	0.5%	1.0%	1.5%
0%		LG0.5	LG1.0	LG1.5
10%	LR10	LR1G0.5	LR1G1.0	LR1G1.5
20%	LR20	LR2G0.5	LR2G1.0	LR2G1.5
30%	LR30	LR3G0.5	LR3G1.0	LR3G1.5

LRG: Epoxy + rubber + graphene nanoplatelets.

**Table 2:** Density of the manufactured composites.

Composition Name	Density (g/cm^3^)	Composition Name	Density (g/cm^3^)	Composition Name	Density (g/cm^3^)
LR10	1.120	LR20	1.115	LR30	1.035
LG0.5	1.145	LG1.0	1.154	LG1.5	1.160
LR1G0.5	1.130	LR2G0.5	1.125	LR3G0.5	1.047
LR1G1.0	1.155	LR2G1.0	1.130	LR3G1.0	1.076
LR1G1.5	1.159	LR2G1.5	1.142	LR3G1.5	1.113

**Table 3:** Surface hardness measurement of the manufactured composites in Shore D scale.

Composition Name	Shore D	Composition Name	Shore D	Composition Name	Shore D
LR10	74.8 ± 0.3	LR20	72.8 ± 0.3	LR30	64.4 ± 0.4
LG0.5	70.6 ± 0.5	LG1.0	76.2 ± 0.4	LG1.5	75.6 ± 0.2
LR1G0.5	74.2 ± 0.4	LR2G0.5	75.8 ± 0.1	LR3G0.5	72.4 ± 0.1
LR1G1.0	72.6 ± 0.2	LR2G1.0	75.0 ± 0.2	LR3G1.0	71.6 ± 0.5
LR1G1.5	74.0 ± 0.5	LR2G1.5	75.8 ± 0.2	LR3G1.5	70.2 ± 0.1

**Table 4:** Three-point bending (3PB) test results.

Composition Name	Ultimate Flexural Stress (MPa)	Flexural Modulus (MPa)	Strain at Break
Neat epoxy	78.96 ± 1.22	1465.83 ± 145.05	0.13 ± 0.017
LG0.5	63.05 ± 11.58	1297.28 ± 193.76	0.064 ± 0.004
LG1.0	74.11 ± 1.78	1439.75 ± 101.60	0.061 ± 0.003
LG1.5	78.97 ± 2.49	1582.23 ± 111.73	0.057 ± 0.004
LR10	61.58 ± 1.64	1454.71 ± 16.28	0.049 ± 0.002
LR20	48.33 ± 1.02	1149.64 ± 20.74	0.045 ± 0.001
LR30	34.27 ± 3.77	478.25 ± 64.13	0.037 ± 0.001
LR1G0.5	59.65 ± 0.54	1478.84 ± 84.15	0.055 ± 0.004
LR1G1.0	59.18 ± 0.20	1475.10 ± 25.70	0.046 ± 0.001
LR1G1.5	59.08 ± 0.67	1294.78 ± 29.11	0.057 ± 0.002
LR2G0.5	47.94 ± 0.25	1417.84 ± 13.69	0.035 ± 0.001
LR2G1.0	48.49 ± 1.01	1474.58 ± 19.79	0.034 ± 0.001
LR2G1.5	48.61 ± 0.74	1537.54 ± 28.97	0.033 ± 0.001
LR3G0.5	35.53 ± 0.55	1064.35 ± 20.10	0.035 ± 0.001
LR3G1.0	36.71 ± 0.42	1079.57 ± 8.66	0.035 ± 0.001
LR3G1.5	38.68 ± 0.24	1135.30 ± 6.65	0.030 ± 0.001

**Table 5:** Parameters used in FEM.

FEM Parameters	
Contact properties	Loading tip—specimen: Frictionless, Hard contact
Mesh properties	C3D8R: A 8-node linear brick
Stress dependence	Isotropic
